# Engineering gene overlaps to sustain genetic constructs *in vivo*

**DOI:** 10.1371/journal.pcbi.1009475

**Published:** 2021-10-08

**Authors:** Antoine L. Decrulle, Antoine Frénoy, Thomas A. Meiller-Legrand, Aude Bernheim, Chantal Lotton, Arnaud Gutierrez, Ariel B. Lindner

**Affiliations:** 1 Université de Paris, INSERM U1001, Paris, France; 2 Université Grenoble Alpes, CNRS UMR5525, Grenoble, France; 3 Université de Paris, INSERM U1284, Center for Research and Interdisciplinarity (CRI), Paris, France; University of Florida, Gainesville, UNITED STATES

## Abstract

Evolution is often an obstacle to the engineering of stable biological systems due to the selection of mutations inactivating costly gene circuits. Gene overlaps induce important constraints on sequences and their evolution. We show that these constraints can be harnessed to increase the stability of costly genes by purging loss-of-function mutations. We combine computational and synthetic biology approaches to rationally design an overlapping reading frame expressing an essential gene within an existing gene to protect. Our algorithm succeeded in creating overlapping reading frames in 80% of *E. coli* genes. Experimentally, scoring mutations in both genes of such overlapping construct, we found that a significant fraction of mutations impacting the gene to protect have a deleterious effect on the essential gene. Such an overlap thus protects a costly gene from removal by natural selection by associating the benefit of this removal with a larger or even lethal cost. In our synthetic constructs, the overlap converts many of the possible mutants into evolutionary dead-ends, reducing the evolutionary potential of the system and thus increasing its stability over time.

## Author summary

Genomes are translated by triplets of nucleotides on two different strands, allowing for six different reading frames. This permits the existence of gene overlaps, often observed in microbial genomes, where two different proteins are encoded on the same piece of DNA, but in different reading frames. Gene overlaps are classically considered an obstacle for both evolution and genetic engineering, as mutations in overlapping regions likely have pleitrotropic effects on several genes. In 2013, we identified specific evolutionary scenarios where the decrease in evolutionary potential caused by gene overlaps could instead be advantageous and selected for. In this work, we demonstrate the use of gene overlaps in another context where reducing evolutionary potential can be useful: preventing evolution from inactivating synthetic circuits. We show that gene overlaps can be engineered to increase the evolutionary stability of genes that are costly to their hosts, by entangling these costly genes with essential genes.

## Introduction

Synthetic biology attempts to use engineering principles to manipulate and reprogram living organisms [1]. This could hold promise for many of the world’s challenges, for example with microbes engineered for bioremediation [2–4] and drugs or fuel biosynthesis [5–7]). Rational genetic engineering poses many design constraints, often dealt with approaches stemming from electrical engineering. However, evolution brings a particular set of challenges [8, 9].

**Fig 1:**
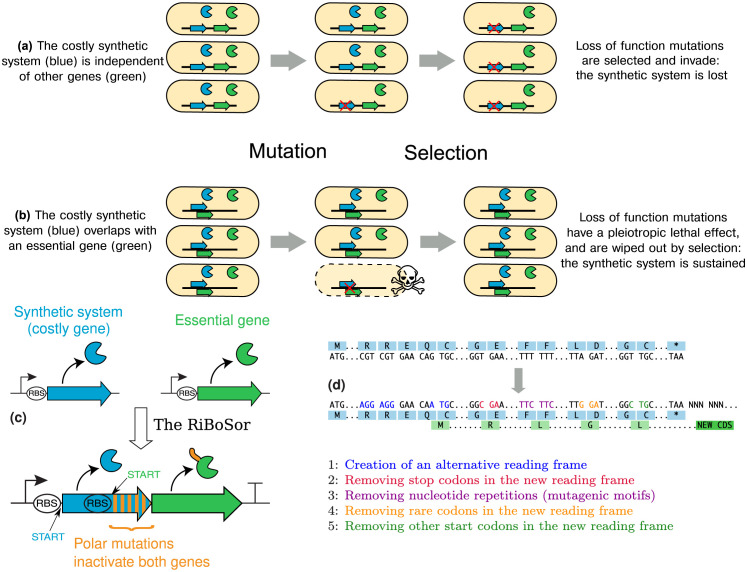
Principle and construction of a Riboverlap. **(a)** Standard case: loss-of-function mutations in the synthetic circuits are selected, because they alleviate the cost of the system. **(b)** The synthetic gene overlaps with an essential gene: loss-of-function mutations are discarded by natural selection because they induce a lethal cost. **(c)** Rational design of a Riboverlap: a new reading frame is created within the gene to protect (blue). An essential gene (green) is cloned downstream, within this reading frame. The CDS of this essential gene is entirely downstream of the pre-existing gene, however the translation initiation motif and thus the beginning of the reading frame lies within the pre-existing gene. The overlapping part (orange) is fused on the 5’ end of the essential protein. **(d)** Examples of synonymous changes made by the algorithm.

Synthetic systems are generally costly to their hosts, and mutants that alter or neutralise them will eventually be selected (Fig 1A). Beyond inactivation by mutations, unforeseen evolution of genetic circuits released in the wild also raises strong concerns. Much research effort is thus dedicated to the restriction of evolutionary potential [10–16]. Similarly, containment and control of engineered organisms outside of the laboratory relies on sophisticated gene circuits that should be made as evolutionary-proof as possible [17–19].

We recently postulated that in nature, coding sequences can evolve overlapping reading frames as a way of reducing their evolvability [20]. Such gene overlaps impose strong constraints on sequences and their evolution [21–24]. If a synthetic gene of interest overlaps with an essential gene, many of the loss-of-function mutations will also affect this essential gene and be evolutionary dead-ends (Fig 1B). This led us [20] and others [8, 25] to suggest that gene overlaps could be engineered and used as a method for preventing gene loss in synthetically engineered organisms.

In this work, we test the use of overlapping reading frames to protect a gene from mutations. We first designed an algorithm to create a new reading frame within an input coding sequence, without modifying the encoded protein. Secondly, we experimentally assessed the evolutionary trajectory of the obtained synthetic constructs by quantifying loss-of-function mutations. We found that overlapping reading frames can be constructed in a large fraction of genes, spanning all bacteria and all functional categories; and that they bring a significant protection from mutations.

These results show that evolutionary constraints can be harnessed to enhance the robustness of systems costly to their host organism. We unveil a promising method to do so, and release our software implementation, the RiBoSor, under a GPL license permitting use and modification by the community.

## Results

### Algorithmic design of Riboverlaps

We developed an algorithm, the RiBoSor, to design an overlapping reading frame within an existing DNA coding sequence (Fig 1C and S1 Fig). This is achieved by creating a translation initiation motif—a ribosome binding site followed by a start codon within the coding sequence, in a different reading frame. The DNA sequence is then further modified (Fig 1D) to make the newly created reading frame suitable for expression of a protein. For example stop codons in the new reading frame are removed with substitutions that are synonymous in the existing reading frame.

An essential gene is cloned downstream of the existing DNA sequence, in the newly created reading frame. The translation of this essential gene thus begins inside the existing gene. The essential protein is N-terminally fused with the end of the original DNA sequence translated in another reading frame. The fused part (orange on Fig 1C) is translated but does not contribute to the protein function. This N-terminal extension is a pure algorithmic result of the location where a translation-initiation motif could be created within the upstream gene. Our method requires that this N-terminal extension does not disturb the folding and function of the downstream protein. The essential gene may be chosen as part of the core genome of the species (in which case the original copy will be removed), or alternatively as an orthogonal, independent gene not present in the core genome but which can be made essential artificially, such as an antibiotic resistance gene.

In theory, the overlap region (orange part on Fig 1C) could span the whole reading frame of the costly gene, although the resulting N-terminal extension may then be more likely to affect function of the downstream protein. In practice, the algorithm searches for the longest possible overlap while minimising non-synonymous mutations occurring in this process and avoiding stop codons and secondary translation initiation sites.

The efficiency of our design (hereafter referred to as Riboverlap) stems from the protection it confers against polar mutations (*i.e*. mutations that affect expression or function of the downstream gene, such as most indels, large rearrangements, and transpositions of insertion sequence). While the overlapped portion of our construct is not *stricto sensu* coding for the essential gene, polar mutations located in this portion will also affect this gene and be purged by natural selection. A large fraction of loss-of-function mutations are thus removed from the mutational pool due to the pleiotropy induced by the Riboverlap.

### Theoretical protective effect of Riboverlaps

The strength of the protection against mutations conferred by such construct depends on several factors. Firstly, the size of the Riboverlap, *i.e*. the fraction of the upstream gene included in the overlapping reading frame (orange part in Fig 1C). Secondly, the proportion of mutations that are frameshifts or other polar mutations such as nonsense mutations. Thirdly, the expected effect of a frameshift mutation compared to the effect of a base pair substitution.

**Fig 2:**
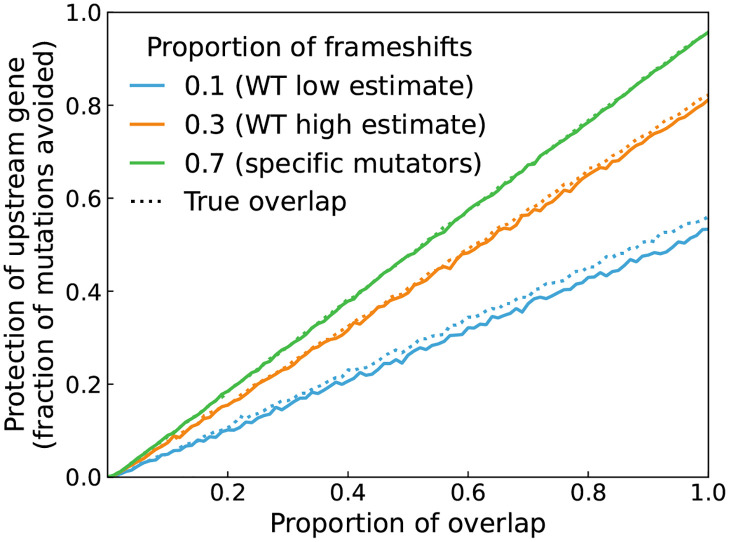
Theoretical protection from mutations conferred by overlapping reading frames. Simulated protection conferred by a gene overlap, depending on the size of the overlap and the fraction of frameshift mutations. The protection is the fraction of loss-of-function mutations in the costly gene that are purged due to the pleiotropic cost induced by the overlap. A true protein overlap (dashed lines) would also protect from non-polar mutations. The average deleteriousness of each amino acid substitutions was *P*_*e*_ = 0.1 (the effect of other values of *P*_*e*_ as well as of alternative models of protein loss-of-function are shown in S2 Fig).

We study the effect of these parameters on the protection of the upstream gene using Monte-Carlo simulations of mutations in a Riboverlap (Fig 2 and S2 Fig). The protection is defined as the fraction of loss-of-function mutations in the costly gene that are counter-selected because of their pleiotropic effect on the essential gene. The parameter range was chosen based on the literature (Methods). Simulation results show that (1) larger overlaps confer a higher protection, (2) the protection is higher when the mutation spectrum is biased towards frameshift, and (3) the protection is higher when a single amino-acid change is less deleterious.

We find that it is possible to achieve up to 90% protection for a full overlap with favourable parameter values (high estimates for the fraction of frameshifts). For smaller overlaps with less favourable parameter values (low fraction of frameshifts notably), the expected protection is smaller but still present.

### Constructing Riboverlaps in bacterial genes

**Fig 3:**
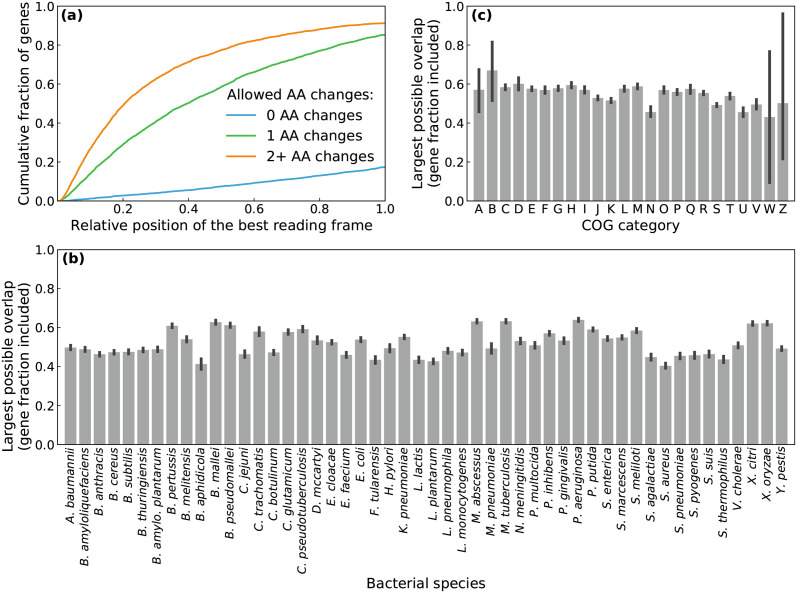
Large overlapping reading frames can be created in many genes. **(a)** Potential Riboverlaps in *E. coli*: cumulative distribution of the first position at which an overlapping reading frames can be created, for all genes of *E. coli* MG1655. Different colors represent different levels of stringency: 0 (blue), 1 (green), or 2 (red) amino acid changes allowed. **(b)** Potential Riboverlaps in other bacterial species: relative size of the largest overlapping frame that can be created. All coding sequences of each organism are averaged, and intermediate level of stringency (1 AA change) was chosen. Error bars represent 95% confidence intervals for all coding sequences of the focal organism. **(c)** Potential Riboverlaps in all COG (Cluster Of Genes) categories. Pooling the 156,542 coding sequences of 50 bacterial species, average relative size of the largest overlapping frame can be created, for each COG category. Error bars represent 95% confidence intervals for all proteins attributed to the focal functional category.

We computed possible Riboverlaps within all coding sequences of *Escherichia coli* using the RiBoSor. The best protection is achieved by the largest overlaps, we thus consider the earliest position at which an overlapping reading frame can be created within each gene. This position of the new reading frame determines the sequence of amino-acids obtained after translation and N-terminally fused to the essential protein. We computed the cumulative distribution of this position, for three different stringency levels of our algorithm, allowing for no, one or several amino acid changes (Fig 3A).

Building a Riboverlap without any amino acid change is only possible in 20% of *E. coli* genes. However, allowing a single change permits to create a Riboverlap in more than 80% of the genes, and a large part of these Riboverlaps (about 65%) have a size higher than half of the gene to be protected. Amino acid substitutions can be necessary for two reasons: creating a translation initiation motif, or removing a stop codon in the newly created frame. Since gram-negative bacteria have a high tolerance to non-consensus ribosome binding sites [26, 27], some of the substitutions suggested by the algorithm to create a translation initiation motif may be dispensable. In addition, some non-synonymous changes do not affect protein function. Since it is increasingly possible to synthesise many variant constructs and experimentally screen their phenotype, allowing one amino acid change is practically feasible.

We also tested the RiBoSor on 49 other model bacterial species, with a comparable success to that of *E. coli* (Fig 3B and S3(A) Fig). Pooling all 156,542 coding sequences of the 50 bacterial species (105,528 unique proteins in the UniProt database), we extracted the 41,177 coding sequences for which we were able to assign a COG (Cluster of Genes) category [28]. We found that it is possible to create Riboverlaps with comparable success for all COG categories (Fig 3C).

Finally, because synthetic circuits often rely on exogenous genes rather than those classically found on bacterial chromosomes, we tested the RiBoSor on 1003 coding sequences found in the iGEM registry of standard biological parts [29]. This registry comprises genes classically used in real-world synthetic circuits, including fluorescent markers, biosynthesis enzymes, transcriptional regulators, and lysis genes. We found that overlapping reading frames can be created in these genes with similar success than in bacterial chromosomes (S3(B) Fig).

### Construction of a Riboverlap *in vivo*

**Fig 4:**
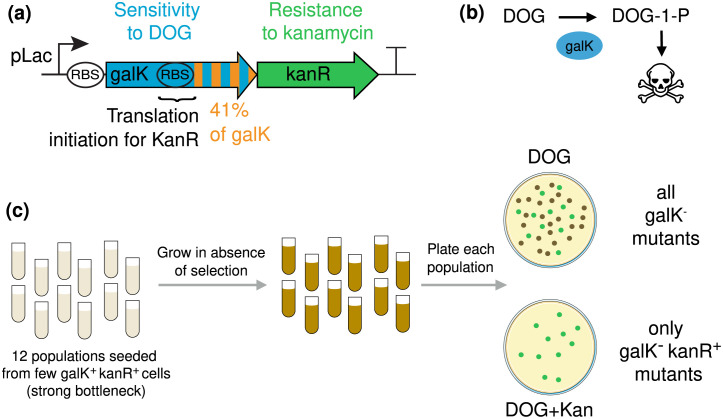
Synthesized construct and experimental design. **(a)** Synthetic construct: overlapping reading frame between galK, which confers sensitivity to DOG, and kanR, which confers resistance to kanamycin. The expression of both genes is controlled by the *pLac* promoter: in absence of the IPTG inducer, the operon is repressed by *lacI^q^*. **(b)** galK metabolises DOG into a toxic compound (see S4 Fig for more details). **(c)** Experimental design: random mutations appear during growth in absence of selection and are subsequently scored by plating on selective medium. This design permits to test whether purifying selection applied on kanR (presence of kanamcyin) protects galK from loss-of-function mutations.

We experimentally tested our design using galK (galactokinase) as a candidate gene to protect. Galactokinase is costly in presence of DOG (2-Deoxy-D-galactose), a galactose analog (Fig 4B and S4 Fig, [30, 31]). The RiBoSor found 9 candidate Riboverlaps in galK, listed in S5 Fig.

We synthesised a candidate Riboverlap that includes 41% of the galK coding sequence, has a perfect consensus translation initiation motif, and does not require any amino acid change (Fig 4A and S5 Fig). As the downstream essential gene, we used a kanamycin resistance gene (kanR) encoding the aminoglycoside phosphotransferase aph(3’)IIIa [32]. Because this is an orthogonal gene rather than an endogenous one, no core genome editing is required. We thus integrate our construct into *E. coli MG1655 Z1 galK-*. Any other gene conditionally or constitutively essential could be used as a downstream gene while keeping the same upstream gene with the same modifications computed by the algorithm.

As a control, we constructed a strain in which galK and kanR share the same promoter but without overlapping reading frame. This permits us to measure the amount of protection conferred by overlapping reading frames compared to the operon strategy previously suggested by Sleigth and collaborators [10]. Both the overlapped construct and the control (operon) could grow on kanamycin and showed sensitivity to DOG, demonstrating that both genes are functional and expressed (S6 Fig). The rate of loss-of-function mutations in galK was found to be similar in the overlapped construct and in the control, showing that our design does not increase mutation rate (S7 Fig).

Both constructs (operon and overlap) are under the control of the *pLac* promoter. Because the background strain expresses the repressor *lacI^q^*, presence of the inducer (IPTG) is necessary for expression of the system.

### Synthetic overlaps protect from mutations

We then test our hypothesis that the overlap with the essential gene (kanR) protects the costly gene (galK) from loss-of-function mutations. In absence of selection in either genes, we quantify the fraction of spontaneous galK loss-of-function mutants that are also kanR loss-of-function mutants. To this end, we use a standard protocol, illustrated in Fig 4C, directly derived from the seminal work of S. Luria and M. Delbrück [33]. Twelve parallel populations are inoculated from a saturated culture after a strong bottleneck (∼20 founder cells). Mutants accumulate without selection during overnight growth in rich medium, in absence of the IPTG inducer. They are scored by plating the twelve populations on DOG alone and DOG with kanamycin at appropriate dilutions. If the overlap is protective, we expect a lower number of colonies on DOG with kanamycin than on DOG alone, as some galK mutants will also lose kanamycin resistance.

We deployed our test in both wild-type and mismatch repair (MMR) defective (Δ*mutS*) strains. The latter hypermutator has a significant frameshift-prone mutation spectrum [34], which allows us to test our hypothesis that the protection is higher when the mutation spectrum is biased toward frameshifts. It is also relevant as often selected in long-term cultures [35]. Many natural isolates of *E. coli* contain mismatch-repair deficient mutants [36, 37], and it has been suggested that this small fraction of mutator bacteria play a major role in adaptive evolution [38]. Furthermore, even in genetically MMR+ populations, it has been suggested that a significant portion of genetic innovations may be due to a subpopulation of cells phenotypically defective for mismatch repair [39].

**Fig 5:**
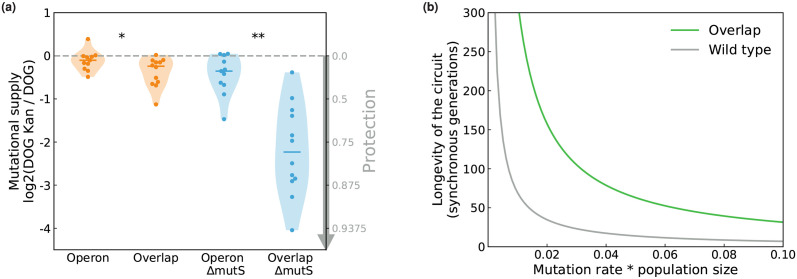
Experimentally measured protection from mutations and expected resulting increase in circuit lifetime. **(a)** The overlap protects galK from loss-of-function mutations. We measure the fraction of galK loss-of-functions (permitting growth on DOG) that did not affect kanR. The lower this fraction, the higher the protection. Each point represents an independent population. The violin plots show the median and kernel density estimation for 12 independent populations. **(b)** Theoretical median lifetime of the circuit, with the measured protection for the Δ*mutS* strain, depending on the rate of loss-of-function mutations and on population size. The lifetime is expressed as the number of generations before the first beneficial loss-of-function mutation emerges.

As shown in Fig 5A, in a wild-type *E. coli*, the implemented overlapping reading frame (covering 41% of the costly gene galK) confers a small protection on the edge of statistical significance threshold (reduction in the fraction of galK mutants that grow on kanamycin compared to the operon control: one-sided Mann-Whitney *U* test, *p* = 0.037, *U* = 95.0). This corresponds to 24% of mutations that are avoided when selecting on kanamycin due to the pleiotropy induced by the overlap. In a Δ*mutS* strain, the protection is stronger and highly significant (*p* = 1.8 × 10^−4^, *U* = 124.0, corresponding to 72% of mutations that are avoided): this is qualitatively consistent with our theoretical prediction that the protection is higher when the mutation spectrum is biased towards frameshifts (Fig 2).

Finally, we estimated the theoretical gain in temporal stability of the synthetic system expected from the measured decrease in mutational supply given by the overlap for the Δ*mutS* strain. The important remaining parameters affecting temporal stability are the rate of loss-of-function mutations in the non-overlapped construct and the population size. We define the circuit lifetime as the time taken for the occurrence of the first loss-of-function mutation that has no deleterious pleiotropic effect and thus that escapes the protection provided by the overlap. The distribution of this time before the occurrence of the first mutation can be calculated analytically (see Methods). We found that the temporal stability of the circuit is predicted to be increased 4–5 folds by the overlap with the measured decrease in mutational supply (Fig 5B).

## Discussion

We show that the evolutionary constraints induced by gene overlaps can be harnessed to design evolutionary robust synthetic systems.

Present in all domains of life, from phages [40] and bacteria [41, 42] to vertebrates [43] including mammals [44], overlapping genes present a fascinating puzzle. They were discovered in the first DNA genome ever sequenced, the phage ΦX174 [40, 45], but the selective pressures and mechanisms leading to their evolution remained elusive for a long time. While neighbouring genes in microbial genomes often overlap by a few base-pair, there are rarer but well-known occurrences of proteins whose coding sequences are significantly or fully included within other genes. This is for example the case for the ASP protein of HIV-1 [46], and the host cell lysis proteins of RNA phage MS2 [47] and DNA phage ΦX174 [40].

Some researchers found this phenomenon so bewildering that they interpreted it as an evidence that ΦX174 was engineered by an extraterrestrial intelligence using a very advanced DNA synthesis technology [48]. Subsequent hypotheses for the evolution of gene overlaps mostly involve genome compression, which can provide several benefits: better encapsulation [49], faster and cheaper replication of the genome [22], and smaller mutational load [50, 51].

Alternatively or as an additional selective force, we suggested [20] that gene overlaps could evolve as an evolvability suppression mechanisms, as defined by Altenberg [52]. Interestingly, it was clear from the very beginning of the study of gene overlaps that an overlapping reading frame is a major constraint on sequence evolution [21]. But to our knowledge, it is only very recently that is has been suggested that this property could be one of the selected features driving the evolution of gene overlaps [20] rather than just an incidental consequence of selection for genome compaction, and that it could be used to restrict the evolutionary potential of engineered circuits [8, 20].

In this work, we specifically implement and test this latter idea. The key specificity of our method is the design of Riboverlaps, where two reading frames overlap but not the actual coding sequences of the two proteins. Complementarily, the Constraining Adaptive Mutations using Engineered Overlapping Sequences (CAMEOS) platform published recently [25] presents a method to engineer a ‘true’ sequence overlap between two proteins. Compared to our approach, CAMEOS increases the entanglement between the two sequences, but as a consequence reduces the chances of successful overlap for any given gene of interest. Blazejewski and collaborators [25] show that overlapping the gene of interest with a toxin using CAMEOS reduces its horizontal transfer to strains lacking the antitoxin, by several orders of magnitude. Taken together, these results confirm with two different approaches that gene overlaps can be engineered to reduce the evolutionary potential of synthetic systems.

This is an important conceptual advance in the field of synthetic biology, which historically relies on engineering-inspired modularity as a design feature. The same way it is a constraint for sequence evolution, the lack of modularity induced by overlapping reading frames is generally seen as a hindrance for genetic engineering. This view for example prompted the refactoring of bacteriophages T7 and ΦX174 without gene overlaps [53, 54]. We follow an alternative approach, showing that a constrained, unmodular design can be an advantage in some situations and can be rationally designed and engineered.

Our design also shares some conceptual similarity with the attempts to make promoters pleiotropic to protect them from downregulatory mutations [10, 11]. Here, beyond the promoter, we protect the coding sequence from the most deleterious classes of mutations: indels and transpositions of insertion sequences.

However, contrarily to the design presented by Blazejewski and collaborators [25], our method does not protect from non-polar substitutions. This implies that it will never provide perfect protection, as seen in Fig 2. Yet the protection is still substantial for large overlaps because the most deleterious mutations are frameshifts. Moreover, we speculate that polar mutations may be more frequent in non-lab conditions than for a wild-type strain in the lab. Transposition of insertion sequences have for example been reported to be prevalent for a synthetic strain in a bioreactor [55], and the rate of indels will be increased if genetic [38] or phenotypic [39] loss of mismatch repair precedes mutations in the system of interest.

Even with a full protein overlap or a favourable mutation spectrum, some mutations neutral for one of the gene but deleterious for the other will eventually arise and invade. This category of methods should thus not be seen as a full protection from mutations, but as tools to reduce the effective mutational supply. Such reduction can make a substantial difference in terms of temporal stability of a synthetic system in a bioreactor as seen in Fig 5B.

Applying our computational method to 105,528 bacterial genes and 1003 iGEM bioparts, we found that there is a large set of genes into which an overlapping reading frame can be created using a consensus translation initiation motif, with no or a single amino acid change. However efficient translation initiation is highly context-dependent due to parameters such as RNA folding [56]. It may be possible to reduce the need for non-synonymous substitutions using computational methods that take into account such parameters [57]. Another limitation of our strategy is the fusion of a protein fragment in the N-terminus of the downstream gene, which may alter the protein function or cause toxicity. This difficulty could be circumvented by the use of a protease to post-translationally cleave the N-terminally fused fragment. Our algorithm could be modified to include a protease recognition domain into the sequence. Alternatively, since the sequence of the fused fragment is calculated by the algorithm, the RiBoSor could be interfaced with any bioinformatic software that predicts features linked to toxicity from the sequence, such as Tango for protein aggregation [58]. Finally, some proteins may be intrinsically more tolerant than others to N-terminal fusion, and the ASKA collection [59] could provide some potential targets. The only other requirement for the downstream gene is the possibility for positive selection. A list of genes matching this criteria is for example provided by LaRossa [60].

In conclusion, our results provide a strong proof of concept that gene overlaps can be rationally engineered to reduce the evolutionary potential of synthetic constructs, that the absence of modularity can be a useful design feature, and that it is possible to rationally design constructs that do not only have a specific phenotype, but also particular evolutionary properties.

## Methods

### Algorithm

The RiBoSor creates a translation initiation motif within the coding sequence of an input gene (the costly gene we want to protect), and makes the new reading frame suitable for cloning and expression of a downstream gene (the essential gene).

This is achieved in several steps. Firstly, each position of the upstream gene is locally analysed to determine whether a translation initiation motif (Shine-Dalgarno sequence followed by 3 to 7 base pairs followed by a start codon) can be created, permitting the opening of an alternative reading frame. Secondly, candidate alternative reading frames are processed to (1) remove STOP codons, (2) remove mononucleotide repeats (that are hotspots for frameshift mutations [61]) and rare codons (that may impede speed and accuracy of expression [62]) when possible, (3) remove other potential translation initiation motifs when possible. Only synonymous changes in the existing reading frame are made by the program, but it can be configured to suggest non-synonymous changes to the user when they are necessary to create a suitable reading frame.

Here we assume that consensus translation initiation motif [63] is the less context dependent motif and will successfully initiate translation in most constructs. The RiBoSor thus attempts to create a Shine-Dalgarno sequence.

### Simulation of the protection

We simulated the occurrence of two types of mutations (base-pair substitutions and frameshift-causing indels) in a construction where two genes (1kb coding sequence for each) overlap by a given fraction (free parameter plotted on the x-axis of Fig 2 and S2 Fig). We aim at quantifying the fraction of mutation in the upstream gene that would be purged by natural selection due to their pleiotropic effect on the downtream gene (effectively quantifying the expected protection of a costly gene stemming from our design).

This requires modelling protein loss-of-function resulting from mutations. We test two different theoretical models (detailed in S2 Fig). For both of them the main parameter (*P*_*e*_) is the average deleteriousness of a single amino-acid change.

Estimations of this parameter are classically reported based on mutagenesis data in the distribution of fitness effects literature. Most of this literature historically focused on RNA viruses, for which the estimates are relatively high and vary from 0.19 [64] or 0.37 [65] to 0.76 [66]. The estimates available for bacteria suggest that the value of *P*_*e*_ would be closer to the lower end of this range [67, 68]. We thus run sets of simulations for different values of *P*_*e*_: 0.1, 0.3, and 0.5 (S2 Fig)).

The last important parameter is *fs*, the proportion mutations that are frameshifts. Estimates in *Escherichia coli* vary between 0.1 and 0.4 for wild type strains, and up to 0.7 or 0.9 for specific frameshift prone mutators such as the Δ*mutS* mismatch repair defective mutant [61]. We thus explore the effect of three different values of *fs*: 0.1, 0.3 and 0.7.

For all combinations of the three aforementioned parameters (the fraction of overlap, the deleteriousness of a single AA substitution *P*_*e*_, and the proportion of mutants that are frameshifts *fs*), we perform 100, 000 Monte-Carlo simulations for each possible size of the Riboverlap, defined as the proportion of the costly gene that is included in the Riboverlap. The output measure of our simulations is the fraction of the mutations impacting the costly gene that would be purged by natural selection because of their impact on the essential gene.

### Screening bacterial genomes

This RiBoSor was run with 3 different levels of stringency: 0, no non-synonymous changes allowed; 1, a single amino acid change allowed; or 2+, a single amino acid change allowed for the creation of the translation initiation motif, and as many as needed for the removal of the stop codons in the new reading frame—the rational being that there are many possible substitutions to remove a stop codon, and thus high chances that one of them is neutral although non-synonymous in the existing reading frame.

50 bacterial species (including *E. coli*) with the highest number of sequenced genomes in RefSeq were chosen for the screen. Computations were parallelized on an HPC platform.

### Strains and growth medium

Both constructs (overlap and control operon) were chromosomally integrated in the *intC* locus of *E. coli* MG1655 Z1 Δ*galK*. The strain Z1 was constructed from MG1655 (CGSC #6300) by transduction (P1vir amplified on DH5alphaZ1) of the Z1 cassette, consisting of constitutively expressed copies of *lacI* and *tetR* and a spectinomycin resistance gene. The Δ*galK* deletion was obtained by P1 transduction from the Keio collection followed by removal of the kanR cassette by FLP recombination using pCP20. The galK-kanR overlap and control operon were constructed using golden gate assembly into plasmids pOverlap and pControl, resp., next to a chloramphenicol cassette and flanked by 50bp *intC* homologies. MG1655 Z1 was first transformed with the pKD46 recombineering helper plasmid and then with PacI-linearized DNA fragments of pOverlap and pControl. Δ*mutS* strains were obtained by P1 transduction of the Δ*mutS* allele from the lab stock.

Stock solutions of the following ingredients are prepared and autoclaved separately: ddH2O, M9x5, CaCl2 at 1M, MgSO4 at 1M, glycerol at 60%v/v, vitamin B1 at 0.1%*w*/*v*, agar at 30g/L. M9 glycerol medium is prepared by mixing 100mL of M9x5, 1.7mL vitamin B1, 1mL MgSO4, 50μL CaCl2, and 3.75mL glycerol with either 393.5mL ddH2O (liquid medium) or 143.5mL ddH2O and 250mL agar (solid medium, the agar is melted and cooled down at 56°C and the other ingredients are heated to 56°C before mixing). 2-Deoxy-D-galactose stock solution was prepared at 20%w/v and stored at 4°C, IPTG stock solution was prepared at 0.5M and stored at −20°C, and kanamycin stock solution was prepared at 100mg/mL and stored at −20°C.

### Phenotype of the constructs and mutants

Both the overlap strain and the operon (control) strain were able to grow in M9 glycerol supplemented with 25μg/mL of kanamycin, while the WT strain (*Escherichia coli* MG1655 Z1 Δ*mutS*) was not. This confirms that kanR is functional (S6 Fig). In M9 glycerol supplemented with 0.2% of DOG, the overlap strain and the operon strain showed no growth after 24 hours at 37°C, while the WT strain could grow (S6 Fig). This confirms that galK is functional and can be used as a costly gene. The concentration of DOG we used was lethal in presence of galK, but could be decreased to modulate the cost. Plating on DOG selects for loss-of-function mutations in galK. We experimentally confirmed that all mutants so obtained loss the ability to grow on galactose minimal medium. We also verified that all colonies obtained on DOG Kan plates are formed of kanamycin resistance cells by growing them in fresh medium containing kanamycin after performing a bottleneck. This excludes the possibility for collective resistance, where non-genetically resistant bacteria could still grow into a colony on the selective plate due to proximity with resistant cells. Tetrazolium chloride can be used to distinguish galK+ and galK- colonies on agar plates [69].

### Mutagenesis protocol

N independent culture tubes containing 5mL of LB are inoculated with 5μL of a 10^−6^ dilution of an overnight culture (approximately 20 founder cells per culture). After 24 hours of growth at 37°C with vigorous shaking at 45° inclination (final density ∼ 4 × 10^9^ cells/mL, corresponding to 28 generations), each culture is plated on two different solid media at appropriate dilutions: on M9 glycerol supplemented with DOG (final concentration 0.2%w/v) and IPTG (final concentration 0.5mM) to count galK loss-of-function mutants, and on the same medium with 25μg/mL kanamycin to count the fraction of these mutants that did not lose kanamycin resistance. The agar plates are incubated 64 hours at 37°C before counting colonies, due to slow growth on M9 glycerol medium. We use M9 glycerol medium to avoid the catabolite repression triggered by glucose [70, 71]. *N* = 11 for the control, and *N* = 12 for the overlap. The reported statistical test compares the fraction of galK- mutants that retained ability to grow on kanamycin, and thus that escape the protection, in the overlap and in the operon control. The rank-biserial correlation *r*, calculated as $1-\frac{2*U}{N_{1}*N_{2}}$, is *r* = −0.44 for the WT constructs and *r* = −0.88 for the Δ*mutS* constructs. We also estimate the number of mutations—which is different from the number of mutants [33]—that escape the protection in the overlap using Jones median estimator [72].

### Temporal stability of the circuit

We compute the median time to the first loss-of-function mutation in the costly system that does not inactivate the essential gene. This time is expressed in number of synchronous generations (total number of divisions divided by constant population size), but this does not imply that generations are synchronous. We assume that the distribution of the number of mutational events of interest in a given unit of time follows a Poisson law whose probability mass function is given by $P\left(m,\Delta t\right)=\frac{e^{-\mu \times N\times \Delta t}\times {\left(\mu \times N\times \Delta t\right)}^{m}}{m!}$, where *m* is the number of mutations, Δ*t* is the number of generations, *μ* is the rate of selectable loss-of-function mutations, and *N* is the population size. The waiting time to the first mutational event then follows an exponential distribution whose complementary cumulative distribution function is given by *P*(0, *t*) = *e*^−*μ*×*N*×*t*^, and thus its median value is $\frac{ln(2)}{u\times N}$. The overlapping reading frame causes a reduction in *μ* because a fraction of loss-of-function mutations induce a pleiotropic fitness cost and are thus not selectable.

These analyses can be extended to the case of multi-genes synthetic circuits. Each brick of the circuit can be separately protected by an overlapping reading frame with a different essential gene. Assuming that the circuit function is lost as soon as one of the gene functions is lost, the probability that the circuit is still functional after *t* generations is given by $p_{survival}\left(t\right)=e^{-N\times t\times \sum_{i=1}^{i=n}\mu_{i}}$, where {*μ*_*i*_} are the rates of selectable loss-of-function mutations in each of the *n* bricks. The overall expected lifetime of a multi-gene circuits with empirically observed parameters (gene size and achievable fraction of overlap) is analysed in S8 Fig.

## Supporting information

S1 Fig The RiBoSor: An algorithm to create alternative reading frames within a gene. The success of the RiBoSor depends on its ability to accurately predict translation initiation motifs. Existing thermodynamic models such as the RBS calculator [57] are not tailored for the evaluation of RBS within coding sequences, and are too slow to screen entire genomes. Furthermore, their use is often restricted to web servers, without availability of the source code or a binary. This increases the complexity of the pipeline and does not guarantee reproducibility and data privacy. We thus use a simpler approximation: we consider that a translation initiation motif is a consensus Shine-Dalgarno sequence [63] followed by 3 to 7 base pairs followed by a START codon. This simplistic criteria ignores important parameters such as secondary structure of messenger RNA [56]. However, since our algorithm proposes several alternative constructs, it is possible to screen the different candidates using a slower and more accurate model, or to directly assay them experimentally. The translation initiation motif of the new reading frame is created using only synonymous changes in the existing reading frame. Considering all possible synonymous variants would lead to a combinatorial explosion: a 300 amino acids sequence (typical *E. coli* protein), has up to 3.2^300^ (≈ 3.3 × 10^50^) possible synonymous variants (worst-case scenario with the average codon redundancy equally distributed, 3.2 is the average number of codons per amino acid), which is well beyond what is computationally feasible. However, finding whether a subsequence can be rewritten to initiate translation is a local problem, only depending on the nucleotides directly surrounding the focal position. We thus apply a local brute-force computation scheme, by considering all the synonymous subsequences in a sliding window of an appropriate size. More specifically, for each position in the input existing gene, the downstream 18 nucleotides (maximal size of the AGGAGG + spacer + START motif: 6+7+3, rounded to the next codon) are examined by a brute-force search algorithm. The algorithm probes all possible combinations of synonymous changes and compares the resulting sequence to the target translation initiation motif. In the worst case, we examine 3.2^18^ possible sequences per position within the input gene, reaching a total of 300 × 3.2^18^ or about 3 × 10^9^ candidate sequences to be evaluated, which is feasible within a reasonable time. Different thresholds can be used to evaluate translation initiation motifs. Since gram-negative bacteria can use variants of the Shine-Dalgarno consensus sequence for translation initiation [27], we consider motifs that have up to one nucleotide substitution relative to this consensus. The algorithm further analyses candidate sequences that match the translation initiation motif. It attempts to introduce synonymous changes in the existing reading frame in order to remove stop codons in the new reading frame and, where possible, to optimise the new reading frame by removing other potential start codons, rare codons—which impede expression [62], and mono-nucleotide repeats longer than three base pairs—which are mutagenic motifs [61]. Since some amino acid substitutions may be neutral, the algorithm can be configured to allow a small number of non-synonymous changes. For example, they may be necessary to remove a stop codon in the new reading frame. Such suppression can be achieved by many different substitutions, and the neutrality of each can be experimentally tested.
(PDF)

S2 Fig Theoretical protection from mutations conferred by overlapping reading frames: Effect of different parameters and models of loss-of-function. Top-left panel is similar to Fig 2. On other panels, we explore the effect of different values for the parameter *P*_*e*_ (deleteriousness of each amino acid substitutions), and different models of protein loss-of-function as a result of amino-acids substitutions. In the discrete stochastic model, a protein can only be either fully functional, or non-functional. Protein loss-of-function resulting from mutations is modelled as a Bernoulli process, assuming that each amino acid change has a probability *P*_*e*_ of turning a functional protein into a non-functional one. The protection conferred by the overlap is then given by the fraction of loss-of-function mutations in the upstream gene that also cause loss-of-function in the downstream gene. In the continuous deterministic model, the activity of a protein is a continuous value between 1 (fully functional) and 0 (no function left). Each amino-acid change affects this activity by a multiplicative factor (1 − *P*_*e*_), giving a formula for the impact of *n* amino-acid substitutions on protein activity: *I*(*n*) = 1 − (1 − *P*_*e*_)^*n*^. The protection conferred by the overlap is then quantified as the dot-product of the impact of the mutations on the downstream and the upstream genes (normalized by their average impact on a single gene).
(PDF)

S3 Fig Potential riboverlaps in 50 bacterial genomes and in iGEM catalog of standard biological parts. **(a)** left panel: full lines represent the average for the 50 bacterial species, and shaded area indicate the standard deviation. **(b)** right panel: full lines represent 1003 coding sequences form iGEM bioparts catalog, and dashed lines represent the pooled 50 bacterial genomes (same data as in left panel, with redundancy removed using UniProt). Similarly to Fig 3A, we run the RiBoSor on all genes of 50 representative bacteria species. These 50 species were chosen as those with the highest number of fully assembled genomes in NCBI database, as a proxy for their popularity in the microbial genomics research field. We found that the RiBoSor is able to create overlapping reading frame in these genes with similar success that for *E. coli MG1655*. Synthetic circuits often use exogenous genes, such as fluorescent reporters, orthogonal transcription factors, or biosynthesis enzymes from other species. To test our computational method on such coding sequences, we parsed the iGEM registry of standard biological parts [29], restricting our search to available protein coding sequences (1349 matches). We conserved those for which the downloaded genbank file actually contains a (single) protein coding sequence, obtaining 1003 protein coding sequences. We run our algorithm on these 1003 sequences, and found that it can create alternative reading frames in these sequences with similar success as in the chromosomal genes of the 50 chosen representative bacterial genomes. This confirms that our method is broadly applicable to endogenous as well as exogenous protein coding sequences, including those classically used in synthetic circuits.
(PDF)

S4 Fig GalK is a counter-selectable gene in presence of DOG. Galactokinase (galK) is costly in a glucose-free growth medium supplemented with 2-Deoxy-D-galactose (DOG) [30, 31]. DOG is an analogue to galactose that can be imported by the same pathway and processed by galK, but can not be further processed by the downstream enzymes of the galactose pathway and will accumulate into toxic intermediates. The expression of a functional galactokinase can be made visible without selection on galactose and amino acids agar medium supplemented with tetrazolium chloride: clones unable to ferment galactose form red colonies [69]. Finally, galK is positively selected in galactose minimal medium. These three properties (possibility of selection, counter-selection and detection in the absence of selection) make galK a gene of choice for our experimental test. We used M9 glycerol as a carbon source for plating on DOG to avoid the catabolite repression triggered by glucose [70, 71]. We experimentally verified that mutants on the DOG plates lost the ability to grow on M9 galactose. Only galK loss-of-function mutations can confer this phenotype (resistance to DOG and inability to grow on galactose). Mutations downstream in the galactose pathway do not prevent the accumulation of deoxy-galactose-1-P. Only the importers are upstream galK, and since they are redundant a single mutation can not prevent the importation of galactose.
(PDF)

S5 Fig Candidates found by the RiBoSor for galK. The algorithm indicates the number of synonymous and non synonymous changes made in the existing gene to create a new reading frame, and the remaining number of non-synonymous changes to be made manually by the experimenter. The remaining changes are not necessary to create a translation initiation motif, but to make the new reading frame suitable for the expression of a downstream coding sequence (eg removal of stop codons in this new reading frame) when no synonymous change permits to achieve this. There are generally several suitable non-synonymous substitutions, and it thus makes sense to let the user manually choose between them. The bold line is the candidate chosen for experimental validation.
(PDF)

S6 Fig Phenotype of the galK-kanR synthetic overlap. The background strain (*E. coli MG1655 Z1 galK-*, orange dots) is sensitive to kanamycin but unaffected by DOG. The overlapping construct (blue dots) and the operon control (green dots) are both resistant to kanamycin and are inhibited by DOG, showing that galK and kanR are functional.
(PDF)

S7 Fig Mutation rate is not affected by the overlap. To assess whether mutation rate of the target sequence is modified by the introduction of an alternative reading frame, we compared the occurrence of galK loss-of function mutations (scored by plating on DOG) in the operon control and in the overlapped construct. We found that mutations in galK seem to happen at a similar rate in both constructs (with or without an overlapping reading frame), and thus that our design does not increase mutation rate.
(PDF)

S8 Fig Predicted temporal stability of a multi-gene circuits. **(a)** Predicted median temporal stability of a n-genes circuit, with or without overlapping reading frames protecting each gene separately. **(b)** Relative increase in temporal stability due to the overlapping reading frame (same data than previous panel). 0 means no increase in stability, 1 means a 100% increase (doubled lifetime). *N* is population size and *μ* is the rate of loss-of-function mutations per base-pair per generation. We estimate the temporal stability of a gene circuit with *n* genes, with or without separate overlapping reading frames protecting each gene. To do so, we use the empirical distribution of possible overlapping reading frames within the 105,528 previously screened protein-coding sequences (Fig 3, allowing 2+ AA changes) and the matching theoretical reduction in mutational pool (Fig 2, *P*_*e*_ = 0.1). For each value of *n*, we ran 10,000 simulations where *n* coding sequences are randomly chosen. Using the size of the *n* sequences and of the largest overlapping reading frame which can be created within each, we compute the median time to the first loss-of-function mutation within the circuit. While the longevity of the circuit expectedly becomes lower when the number of genes is higher, the relative increase in longevity confered by the overlapping reading frames remains constant. This shows that our method can scale to multi-gene circuits.
(PDF)
