# Supplementary material for: Engineering gene overlaps to sustain genetic constructs in vivo
Source: PLoS Comput Biol. 2021 Oct 8;17(10):e1009475. doi: 10.1371/journal.pcbi.1009475 (PMC8528312; doi:10.1371/journal.pcbi.1009475)
Supplement: S1 Fig — The success of the RiBoSor depends on its ability to accurately predict translation initiation motifs. Existing thermodynamic models such as the RBS calculator [57] are not tailored for the evaluation of RBS within coding sequences, and are too slow to screen entire genomes. Furthermore, their use is often restricted to web servers, without availability of the source code or a binary. This increases the complexity of the pipeline and does not guarantee reproducibility and data privacy. We thus use a simpler approximation: we consider that a translation initiation motif is a consensus Shine-Dalgarno sequence [63] followed by 3 to 7 base pairs followed by a START codon. This simplistic criteria ignores important parameters such as secondary structure of messenger RNA [56]. However, since our algorithm proposes several alternative constructs, it is possible to screen the different candidates using a slower and more accurate model, or to directly assay them experimentally. The translation initiation motif of the new reading frame is created using only synonymous changes in the existing reading frame. Considering all possible synonymous variants would lead to a combinatorial explosion: a 300 amino acids sequence (typical E. coli protein), has up to 3.2300 (≈ 3.3 × 1050) possible synonymous variants (worst-case scenario with the average codon redundancy equally distributed, 3.2 is the average number of codons per amino acid), which is well beyond what is computationally feasible. However, finding whether a subsequence can be rewritten to initiate translation is a local problem, only depending on the nucleotides directly surrounding the focal position. We thus apply a local brute-force computation scheme, by considering all the synonymous subsequences in a sliding window of an appropriate size. More specifically, for each position in the input existing gene, the downstream 18 nucleotides (maximal size of the AGGAGG + spacer + START motif: 6+7+3, rounded to the next codon) [file pcbi.1009475.s001.pdf]

```
for i ∈ [1, len(Gene1)/3] do
    // Sliding window: condons C_i to C_{i+5}
    for (S_0, ..., S_5) ∈ ∏_{j∈[0,5]} synonymous(C_{i+j}) do
        if ContainsRBSspacerSTART(S_0S_1S_2S_3S_4S_5)
         then
            newsequence=C_1..C_{i-1}S_1..S_6C_{i+6}..C_n;
            PosSTART=3 * i + PosStartInMotif;
            // Making only synonymous changes in
               gene1
            RemoveSTOPS(&newsequence, PosSTART);
            RemoveOtherRBS(&newsequence,
             PosSTART);
            RemoveFShotspots(&newsequence);
            ReportCandidateSequence(newsequence);
        end
    end
end
```
