# Supplementary figures and images for: Engineering gene overlaps to sustain genetic constructs in vivo
Source: PLoS Comput Biol. 2021 Oct 8;17(10):e1009475. doi: 10.1371/journal.pcbi.1009475 (PMC8528312; doi:10.1371/journal.pcbi.1009475)

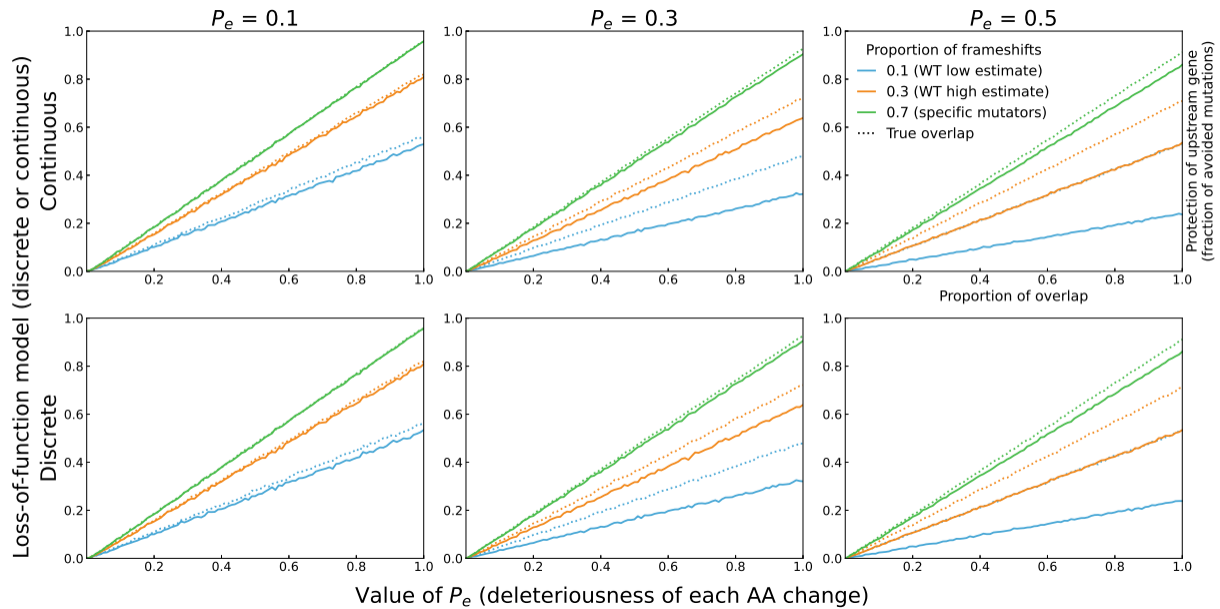

Supplement: S2 Fig — Top-left panel is similar to Fig 2. On other panels, we explore the effect of different values for the parameter Pe (deleteriousness of each amino acid substitutions), and different models of protein loss-of-function as a result of amino-acids substitutions. In the discrete stochastic model, a protein can only be either fully functional, or non-functional. Protein loss-of-function resulting from mutations is modelled as a Bernoulli process, assuming that each amino acid change has a probability Pe of turning a functional protein into a non-functional one. The protection conferred by the overlap is then given by the fraction of loss-of-function mutations in the upstream gene that also cause loss-of-function in the downstream gene. In the continuous deterministic model, the activity of a protein is a continuous value between 1 (fully functional) and 0 (no function left). Each amino-acid change affects this activity by a multiplicative factor (1 − Pe), giving a formula for the impact of n amino-acid substitutions on protein activity: I(n) = 1 − (1 − Pe)n. The protection conferred by the overlap is then quantified as the dot-product of the impact of the mutations on the downstream and the upstream genes (normalized by their average impact on a single gene). (PDF) [file pcbi.1009475.s002.pdf]

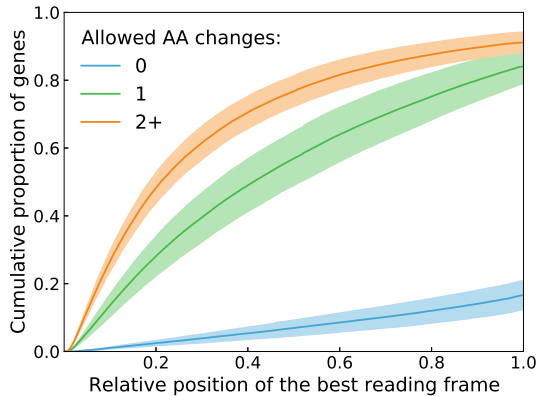

(a) 50 representative bacterial genomes

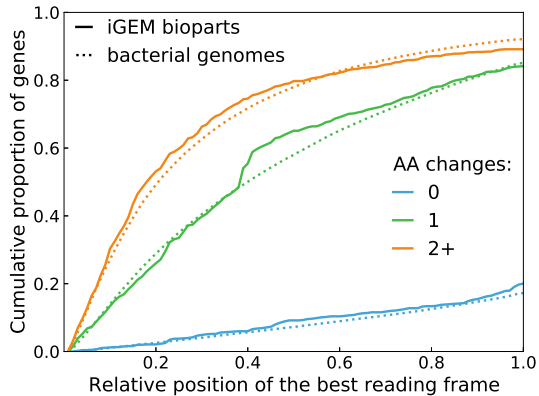

(b) iGEM bioparts

Supplement: S3 Fig — (a) left panel: full lines represent the average for the 50 bacterial species, and shaded area indicate the standard deviation. (b) right panel: full lines represent 1003 coding sequences form iGEM bioparts catalog, and dashed lines represent the pooled 50 bacterial genomes (same data as in left panel, with redundancy removed using UniProt). Similarly to Fig 3A, we run the RiBoSor on all genes of 50 representative bacteria species. These 50 species were chosen as those with the highest number of fully assembled genomes in NCBI database, as a proxy for their popularity in the microbial genomics research field. We found that the RiBoSor is able to create overlapping reading frame in these genes with similar success that for E. coli MG1655. Synthetic circuits often use exogenous genes, such as fluorescent reporters, orthogonal transcription factors, or biosynthesis enzymes from other species. To test our computational method on such coding sequences, we parsed the iGEM registry of standard biological parts [29], restricting our search to available protein coding sequences (1349 matches). We conserved those for which the downloaded genbank file actually contains a (single) protein coding sequence, obtaining 1003 protein coding sequences. We run our algorithm on these 1003 sequences, and found that it can create alternative reading frames in these sequences with similar success as in the chromosomal genes of the 50 chosen representative bacterial genomes. This confirms that our method is broadly applicable to endogenous as well as exogenous protein coding sequences, including those classically used in synthetic circuits. (PDF) [file pcbi.1009475.s003.pdf]

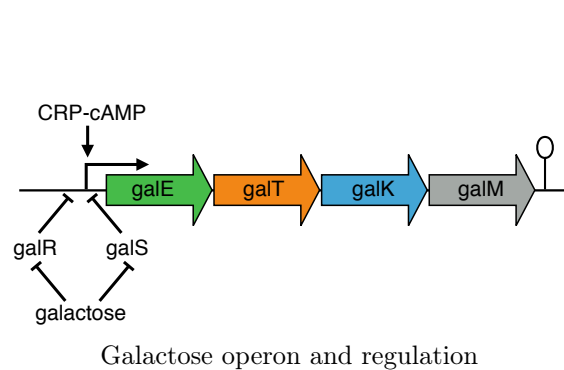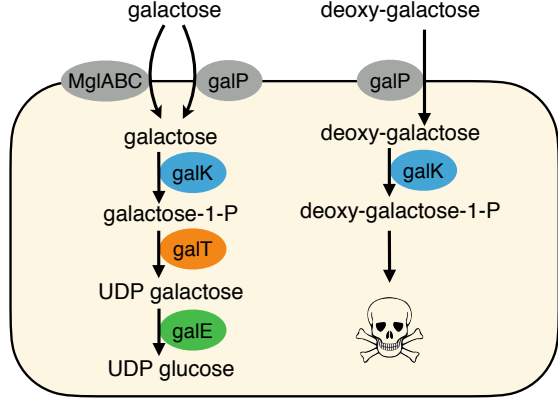

Metabolism of galactose and 2-Deoxy-D-galactose

Supplement: S4 Fig — Galactokinase (galK) is costly in a glucose-free growth medium supplemented with 2-Deoxy-D-galactose (DOG) [30, 31]. DOG is an analogue to galactose that can be imported by the same pathway and processed by galK, but can not be further processed by the downstream enzymes of the galactose pathway and will accumulate into toxic intermediates. The expression of a functional galactokinase can be made visible without selection on galactose and amino acids agar medium supplemented with tetrazolium chloride: clones unable to ferment galactose form red colonies [69]. Finally, galK is positively selected in galactose minimal medium. These three properties (possibility of selection, counter-selection and detection in the absence of selection) make galK a gene of choice for our experimental test. We used M9 glycerol as a carbon source for plating on DOG to avoid the catabolite repression triggered by glucose [70, 71]. We experimentally verified that mutants on the DOG plates lost the ability to grow on M9 galactose. Only galK loss-of-function mutations can confer this phenotype (resistance to DOG and inability to grow on galactose). Mutations downstream in the galactose pathway do not prevent the accumulation of deoxy-galactose-1-P. Only the importers are upstream galK, and since they are redundant a single mutation can not prevent the importation of galactose. (PDF) [file pcbi.1009475.s004.pdf]

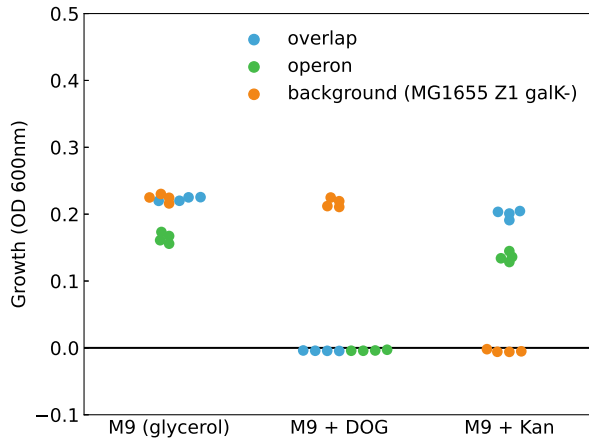

Supplement: S6 Fig — The background strain (E. coli MG1655 Z1 galK-, orange dots) is sensitive to kanamycin but unaffected by DOG. The overlapping construct (blue dots) and the operon control (green dots) are both resistant to kanamycin and are inhibited by DOG, showing that galK and kanR are functional. (PDF) [file pcbi.1009475.s006.pdf]

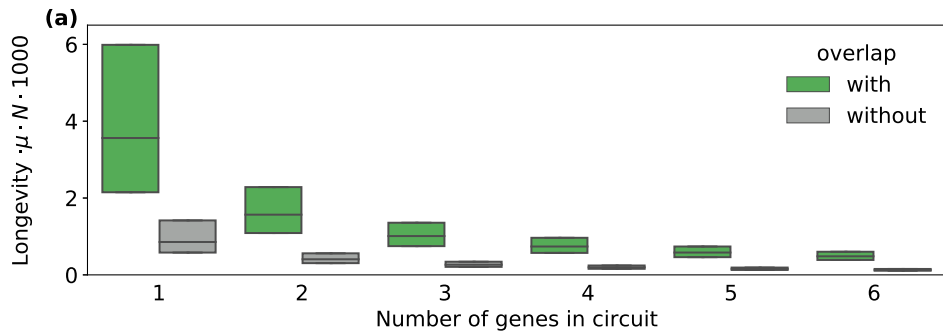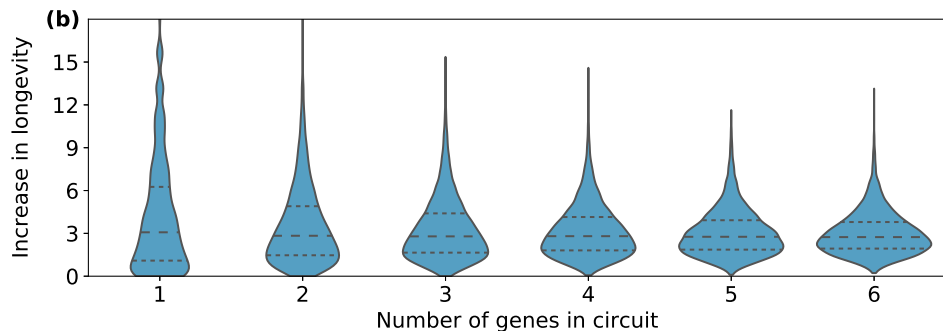

Supplement: S8 Fig — (a) Predicted median temporal stability of a n-genes circuit, with or without overlapping reading frames protecting each gene separately. (b) Relative increase in temporal stability due to the overlapping reading frame (same data than previous panel). 0 means no increase in stability, 1 means a 100% increase (doubled lifetime). N is population size and μ is the rate of loss-of-function mutations per base-pair per generation. We estimate the temporal stability of a gene circuit with n genes, with or without separate overlapping reading frames protecting each gene. To do so, we use the empirical distribution of possible overlapping reading frames within the 105,528 previously screened protein-coding sequences (Fig 3, allowing 2+ AA changes) and the matching theoretical reduction in mutational pool (Fig 2, Pe = 0.1). For each value of n, we ran 10,000 simulations where n coding sequences are randomly chosen. Using the size of the n sequences and of the largest overlapping reading frame which can be created within each, we compute the median time to the first loss-of-function mutation within the circuit. While the longevity of the circuit expectedly becomes lower when the number of genes is higher, the relative increase in longevity confered by the overlapping reading frames remains constant. This shows that our method can scale to multi-gene circuits. (PDF) [file pcbi.1009475.s008.pdf]
