# Supplementary material for: Engineering gene overlaps to sustain genetic constructs in vivo
Source: PLoS Comput Biol. 2021 Oct 8;17(10):e1009475. doi: 10.1371/journal.pcbi.1009475 (PMC8528312; doi:10.1371/journal.pcbi.1009475)
Supplement: S5 Fig — The algorithm indicates the number of synonymous and non synonymous changes made in the existing gene to create a new reading frame, and the remaining number of non-synonymous changes to be made manually by the experimenter. The remaining changes are not necessary to create a translation initiation motif, but to make the new reading frame suitable for the expression of a downstream coding sequence (eg removal of stop codons in this new reading frame) when no synonymous change permits to achieve this. There are generally several suitable non-synonymous substitutions, and it thus makes sense to let the user manually choose between them. The bold line is the candidate chosen for experimental validation. (PDF) [file pcbi.1009475.s005.pdf]

Start pos.	Frame	Synonymous changes	Non synonymous	Remaining
475	1	96	1	1
544	1	91	1	1
**675**	**2**	**74**	**0**	**0**
742	1	75	1	1
995	1	61	1	0
998	1	62	1	0
1099	1	56	1	0
1129	2	31	1	0
1130	2	29	1	0
