# Supplementary material for: Engineering gene overlaps to sustain genetic constructs in vivo
Source: PLoS Comput Biol. 2021 Oct 8;17(10):e1009475. doi: 10.1371/journal.pcbi.1009475 (PMC8528312; doi:10.1371/journal.pcbi.1009475)
Supplement: S7 Fig — To assess whether mutation rate of the target sequence is modified by the introduction of an alternative reading frame, we compared the occurrence of galK loss-of function mutations (scored by plating on DOG) in the operon control and in the overlapped construct. We found that mutations in galK seem to happen at a similar rate in both constructs (with or without an overlapping reading frame), and thus that our design does not increase mutation rate. (PDF) [file pcbi.1009475.s007.pdf]

Construct	mutational events	95% CI lower	95% CI upper	p-value
MGZ1 control	397.255	354.500	441.700	} 0.85
MGZ1 overlap	357.154	319.100	396.800	
MGZ1 mutS- control	27.347	20.737	34.611	} 0.71
MGZ1 mutS- overlap	29.866	23.115	37.237	
